# Rate-Dependent Hysteresis Modeling and Displacement Tracking Control Based on Least-Squares SVM for Axially Pre-Compressed Macro-Fiber Composite Bimorph

**DOI:** 10.3390/ma15186480

**Published:** 2022-09-18

**Authors:** Kaiming Hu, Hujian Ge, Hua Li, Shenglong Xie, Suan Xu

**Affiliations:** 1 School of Mechanical and Electrical Engineering, China Jiliang University, Hangzhou 310018, China; 2 Key Laboratory of Intelligent Manufacturing Quality Big Data Tracing and Analysis of Zhejiang Province, China Jiliang University, Hangzhou 310018, China; 3 School of Aeronautics and Astronautics, Zhejiang University, Hangzhou 310027, China; 4 Key Laboratory of Soft Machines and Smart Devices of Zhejiang Province, Hangzhou 310027, China

**Keywords:** rate-dependent hysteresis modeling, displacement tracking, axially pre-compressed bimorph, macro-fiber composite, least-squares support vector machine, feedforward plus feedback control

## Abstract

The new axially pre-compressed macro-fiber composite bimorph (MFC-PBP) can produce large displacement and output power. However, it has the property of strong rate-dependent hysteresis nonlinearity, which challenges the displacement tracking control of morphing structures. In this paper, the least-squares support vector machine (LS-SVM) is applied to model the rate-dependent hysteresis of MFC-PBP. Compared with the predicated results of the series model of the Bouc–Wen model and Hammerstein model (BW-H), the LS-SVM model achieved higher predication accuracy and better generalization ability. Based on the LS-SVM hysteresis compensation model, with the support vector pruning, the displacement tracking feedforward compensator is obtained. In order to improve the displacement tracking accuracy, the LS-SVM feedforward compensator combined with the proportional and integral (PI) controller and the feedforward plus feedback control experiment is carried out on the displacement tracking of MFC-PBP. The test results show that the feedforward plus feedback displacement tracking control loop based on the LS-SVM model also has a higher displacement tracking accuracy than that based on the inverse model of BW-H.

## 1. Introduction

**Figure 1:**
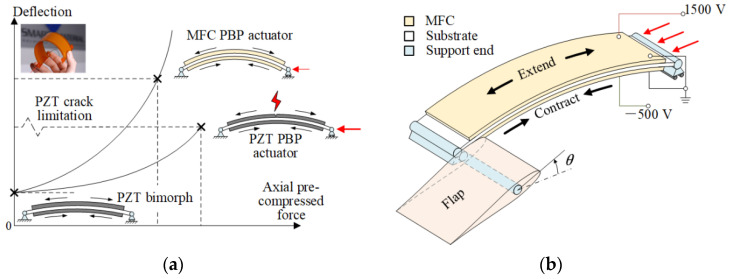
Operating principle of MFC-PBP actuator: (**a**) comparison of actuation performances of MFC-PBP and PZT-PBP; (**b**) micro flap derived by MFC-PBP [5].

The piezoelectric bimorph has the characteristics of fast response and small volume, which is suitable for driving the flap deflection of a micro aerial vehicle (MAV). However, the small deformation and output power limit the application of the bimorph, so the concept of pre-compressed piezoelectric bimorph (PBP) was proposed by Barrett et al. [1,2]. The bimorph employs the axial pressure to reduce its equivalent bending stiffness. Compared with the bimorph without axial pressure, PBP could increase the output displacement by more than three times and maintain the output force, thus the output power is significantly increased. In addition, Barrett et al., applied the PBP to various types of MAVs [3]. It is well known that lead zirconate titanate ceramics (PZT) have difficulty enduring large tensile loads, while the piezoelectric macro-fiber composite (MFC) composed of PZT fiber layer, electrodes, and polyimide film [4] has some tensile resistance ability, and it is more flexible. Thus, the axially pre-compressed MFC bimorph (MFC-PBP) was proposed by Hu et al., and they carried out the theoretical and experimental research [5,6]. The results show that the MFC-PBP can produce larger output displacement and output power than those of the original PBP, as shown in Figure 1.

In addition to the output work, the control accuracy of the actuator is another important aspect of the actuator performance. Even if the actuator meets the requirements of deflection and output power, while the control accuracy is low, it is still difficult to be used as a reliable flap actuator for MAVs. Bilgen et al. [7] conducted many test flights of fixed-wing MAVs with the MFC driving flight control surface deformation, but the MAVs often crashed due to control system failure. They attributed the reason to not considering the hysteresis compensation of MFCs in the control system design. Trabia et al. [8] studied the small morphing wing deflected by the MFC bimorph, and found that the controller based on the actuator linear model can meet the requirements for tracking constant or slowly changing displacement commands; however, the hysteresis nonlinearity of the actuator must be considered in the controller design for the rapidly changing displacement commands. Thus, the hysteresis of MFC actuators can seriously affect the accuracy of the flight control system of MAVs.

For accurately describing the hysteresis nonlinearity in the piezoelectric actuators and achieving the fast and high precision displacement tracking control, scholars have carried out many studies [9,10,11]. Thus far, existing hysteresis models can be divided into two categories: physical models and phenomenological models [12,13]. The physical model has a clear physical meaning, but the form is complex and the universality is low [14], and while the phenomenological models are without clear physical meaning, their model forms are relatively simple and can be easily applied to various hysteresis phenomena; therefore, phenomenological models are more widely used than physical models [13,15]. Zheng et al., and Chen et al., applied the Preisach model to describe the hysteresis of MFC [16,17]. Yang et al., employed the Bouc–Wen approach to model and control the hysteresis of MFC unimorph [18]. However, the hysteresis of a piezoelectric actuator is also affected by the variation rate of the driving voltage. The abovementioned operator and differential equation phenomenological hysteresis models have weak rate-dependent hysteresis description ability [19]. Intelligent models such as artificial neural network (ANN) and support vector machine (SVM) can describe the rate-dependent characteristics and avoid solving the inverse model [10]. Cheng et al., used multilayer ANN to realize the hysteresis compensation and the displacement tracking of the piezoelectric micro-displacement platform [20]. However, ANN has problems of a large number of preset parameters and is easy to fall into local optimality; SVM can avoid these problems. Xu et al. [21] employed the least-squares SVM (LS-SVM) to model and control the hysteresis of the piezoelectric platform and obtain the high prediction accuracy and displacement tracking accuracy [21]. In order to reduce the influence of outliers, the weighted LS-SVM (WLSSVM) was proposed by Suykens et al. [22]. To further reduce the redundancy of WLS-SVM and optimize the weighting strategy, a new adaptive WLS-SVM (AWLS-SVM) was proposed by Mao et al. [23]. Moreover, Kuh et al. [24] proposed the method of support vector pruning to reduce the model complexity and improve the compensation accuracy, which are more suitable for real-time application [25].

**Figure 2:**
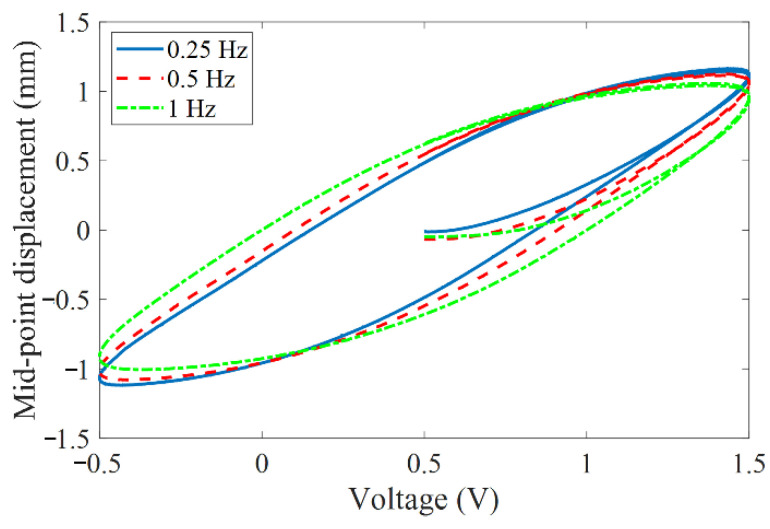
Hysteresis loop of MFC-PBP under different frequency excitation voltages.

In addition, most of the hysteresis models and control methods were established for the piezoelectric stack or the piezoelectric stack as the driving source of the precision actuation system. Their action strokes are relatively small (10~200 μm) and the hysteresis degrees are also relatively light (10–15%) [18,20]. The nonlinear hysteresis in these cases is mainly caused by the non-180° turn of the ferroelectric domain and its incomplete reversibility in piezoelectric ceramic [26]. However, MFC-PBP has a large output displacement, which includes not only the hysteresis caused by the non-180° turn of the ferroelectric domain, but also the creep characteristics of the polymer materials (Kapton) and the nonlinear constituent of the MFC in the large tensile state [6,27]. Thus, the remarkable rate-dependent hysteresis will appear in the case of high deformation. As shown in Figure 2, the mid-point displacement hysteresis loop of 55 mm long simply supported MFC-PBP measured under different frequency excitation voltages.

Thus far, there is less research on the hysteresis modeling and control for the piezoelectric unimorph and bimorph under pre-stress. The sliding mode hysteresis control of the THUNDER actuators based on model prediction was carried out by Kim et al. [28]. The hysteresis linearization control of feedforward plus feedback for the PBP was carried out by Hu et al. [29], where the displacement tracking loop was composed by the compensator based on the inverse model of a BW-H (series model of Bouc–Wen model and Hammerstein model) model and an adaptive PID (proportional–integral–differential) controller, but the displacement tracking accuracy needs to be further improved.

Thus, this paper employs the LS-SVM to model the rate-dependent hysteresis nonlinearity of the MFC-PBP. We compare the LS-SVM the BW-H model to verify the evaluation accuracy of LS-SVM. Then, the feedforward LS-SVM hysteresis compensator and the feedback PI (proportional–integral) controller are combined, so as to realize the high-precision tracking control for the various complex large displacement commands.

## 2. Rate-Dependent Hysteresis Modeling

This section applies the following two phenomenological models of BW-H and LS-SVM to model the rate-dependent hysteresis of the MFC-PBP and compares their modeling accuracy and generalization ability. The BW-H model and LS-SVM model represent the traditional differential model and the artificial intelligence network model, respectively.

### 2.1. Measurements Systems for Rate-Dependent Hysteresis

**Figure 3:**
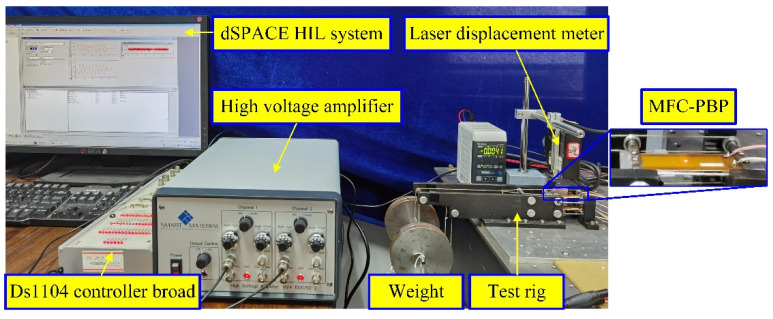
Test platform for MFC-PBP.

Since both models are data-driven, the hysteresis loops of the MFC-PBP need to be firstly measured by experiment. The test platform for MFC-PBP is shown in Figure 3.

The boundary condition of MFC-PBP is that one end can rotate, and the other end can slide and rotate. The type of MFC is M-4010 P1, the thickness of the aluminum substrate sheet is 0.3 mm, the length is 55 mm, and the width is 16 mm. The adhesive layer between the aluminum substrate sheet and MFC film is Loctite Hysol E-120HP. A laser displacement meter (Keyence LK-G30) was used to measure the mid-point transverse displacement of MFC-PBP. A dSPACE DS1104 controller board was used to collect and process the test data for measuring and controlling the deformation of MFC-PBP. The control computer memory is 8 GB, and the processor is Intel Core2 E8500. For tracking the high-frequency displacement signal, HVA 1500/50-2 high voltage and large bandwidth power amplifier was applied to supply the driving voltages and powers for MFC-PBP. The imposed axial pre-compressed force on the MFC-PBP is 18.7 N.

### 2.2. Series Model of Bouc–Wen Model and Hammerstein Model

**Figure 4:**
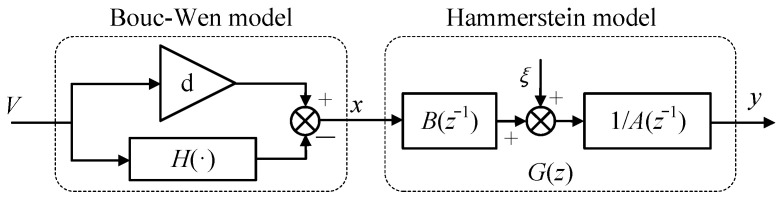
Block diagram of BW-H model.

Since the Bouc–Wen model has weak rate-dependent description ability, the BW-H model is employed in this paper, which is made in a series of the Bouc–Wen differential model and the Hammerstein discrete transfer function [29,30]. The Bouc–Wen model describes the hysteresis nonlinearity in the quasi-static conditions. The Hammerstein model is an autoregressive model of discrete transfer function, which describes the rate-dependent ability. The block diagram of the BW-H is shown in Figure 4.

The BW-H model can be expressed as follows

(1a) $$\{\begin{array}{c} x\left(t\right)=X\left(t\right)-H\left(t\right)=dV\left(t\right)-H\left(t\right)\\ \dot{H}\left(t\right)=\alpha \dot{V}\left(t\right)-\beta \left|\dot{V}\left(t\right)\right|{\left|H\left(t\right)\right|}^{n-1}H\left(t\right)-\gamma \dot{V}\left(t\right){\left|H\left(t\right)\right|}^{n} \end{array}$$

(1b) y(t)=x(t)G(z)

where (1a) is the Bouc–Wen differential model, and the model treats the hysteresis curve as a superposition of the linear component and the hysteresis component. *X*(*t*) is the linear displacement component, *H*(*t*) is the hysteresis displacement component, *V*(*t*) is the excitation voltage, *d* is the proportional coefficient between the linear displacement component and the excitation voltage, and *α*, *β*, *γ*, and n are the shape control parameters of hysteresis loop. In Figure 4, *x*(*t*) is not only the output of the static hysteresis model, but also the input of the dynamic linear module *G*(*z*); ξ is the noise; *G*(*z*) = *B*(z^−1^)/*A*(*z*^−1^) is a discrete transfer function. The relationship between *A*(*z*^−1^) and *B*(*z*^−1^) can be expressed as

(2) $$A\left(z^{-1}\right)y_{k}=B\left(z^{-1}\right)x_{k}+\xi_{k}$$

where *z*^−1^ represents one sample period delay in the discrete time series, the subscript *k* represents the variables at the *k*-th time step, and the expressions of *A*(*z*^−1^) and *B*(*z*^−1^) can be expressed as

(3) $$\{\begin{array}{c} A\left(z^{-1}\right)=1+a_{1}z^{-1}+a_{2}z^{-2}+\cdots +a_{n}z^{-n}\\ B\left(z^{-1}\right)=b_{0}z^{-1}+b_{1}z^{-2}+\cdots +b_{m}z^{-m} \end{array}$$

where *a*_1…n_ and *b*_0…m_ are the undetermined coefficients, which can be determined by the least square parameter identification.

### 2.3. LS-SVM Regression Model

The LS-SVM regression model can be expressed as follows

(4) $$y_{k}=f\left(x_{k}\right)+\xi_{k}$$

where ***x****_k_* is the input signal, *f* ( ) is a one-to-one mapping nonlinear regression function, *y*_k_ is the output, and *ξ_k_* is the regression error at the *k*-th time step.

For converting the multi-valued mapping of hysteresis into the one-to-one mapping, the model input is set as follows:

(5) $$x_{k}=\left[u_{k}\;u_{k-1}\ldots u_{k-p}\;y_{k-1}\ldots y_{k-q}\right]$$

where *u_k_* and *y_k_*_−1_ are the input voltage at the *k*-th time step and output displacement at the (*k* − 1)-th time step, respectively; *p* and *q* are the system orders.

Nonlinear regression function *f* ( ) can be expressed as follows:

(6) $$f\left(x\right)=w^{T}\varphi \left(x\right)+b$$

where w is the weight vector, *φ*( ) is the nonlinear mapping function, and *b* is the scalar bias; w and *b* can be obtained by solving the following optimal problems:

(7a) $$\underset{\omega ,\xi ,b}{\min}J\left(w,\xi \right)=\frac{1}{2}w^{T}w+\frac{C}{2}\overset{N}{\underset{k=1}{\sum }}\xi_{k}^{2}$$

(7b) $$s.t.y_{k}=w^{T}\varphi \left(x_{k}\right)+b+\xi_{k}$$

where *C* is a positive real constant which balances the error and model complexity. For solving this high-dimension optimization problem, the optimal problem (7) can be converted into the form of Lagrange function as follows:

(8) $$L\left(w,b,\xi ,\alpha \right)=J\left(w,\xi \right)-\overset{N}{\underset{k=1}{\sum }}\alpha_{k}\left[w^{T}\varphi \left(x_{k}\right)+b+\xi_{k}-y_{k}\right]$$

where *α_k_*_=1…N_ are the Lagrange multipliers. The optimal solution satisfies the following conditions:

(9) $$\{\begin{array}{c} \frac{\partial L}{\partial w}=0\to w=\overset{N}{\underset{k=1}{\sum }}\alpha_{k}\varphi \left(x_{k}\right)\\ \frac{\partial L}{\partial \xi_{k}}=0\to \alpha_{k}=C\xi_{k}\\ \frac{\partial L}{\partial b}=0\to \overset{N}{\underset{k=1}{\sum }}\alpha_{k}=0\\ \frac{\partial L}{\partial \alpha_{k}}=0\to w^{T}\varphi \left(x_{k}\right)+b+\xi_{k}-y_{k}=0 \end{array}$$

Removing the w and *ξ_k_* in the above formula yields the following equation:

(10) $$\left[\begin{array}{cc} 0 & e_{N\times 1}^{T}\\ e_{N\times 1} & \Omega +I_{N}/C \end{array}\right]\left[\begin{array}{c} b\\ \alpha \end{array}\right]=\left[\begin{array}{c} 0\\ Y \end{array}\right]$$

where ***e***_N×1_ = [1 1 …1]^T^, ***α*** = [*α*_1_ *α*_2_…*α*_N_]^T^, and ***Y*** = [*y*_1_ *y*_2_…*y*_N_]^T^; ***I***_N_ is an N-dimension identity matrix; $\Omega_{\mathrm{ij}}=\varphi^{T}\left(x_{i}\right)\varphi \left(x_{j}\right)=K\left(x_{i},x_{j}\right)$; *K* is the kernel function, and here *K* is the radial basic function as follows:

(11) $$K\left(x_{i},x_{j}\right)=\exp\left(-\frac{||x_{i}-x_{j}||{}^{2}}{2\sigma^{2}}\right)$$

where ***σ*** is the kernel width parameter.

Based on ***α*** and *b* obtained by (10), the LS-SVM regression model can be expressed as

(12) $$y\left(x\right)=\overset{N}{\underset{k=1}{\sum }}\alpha_{k}K\left(x_{k},x\right)+b$$

## 3. Model Parameter Identification and Model Comparison

The purpose of modeling is to identify the parameters of the model and realize the high precision regression of the nonlinear hysteresis model. Thus, this section uses the genetic algorithm (GA) and least-squares estimation to identify the parameters of the BW-H model, and apply the LS-SVMlab1.8 toolbox to build the LS-SVM model. Then, based on these identified models, the modeling precision and generalization ability of both models are verified by a multiple frequency superposition signal.

### 3.1. Parameters Identification of BW-H Model

Firstly, the quasi-static hysteresis displacement is measured to identify the parameters of the Bouc–Wen model, and the expression of the quasi-static excitation voltage signal is as follows:

(13) u=1000sin(0.5πt)+500

**Figure 5:**
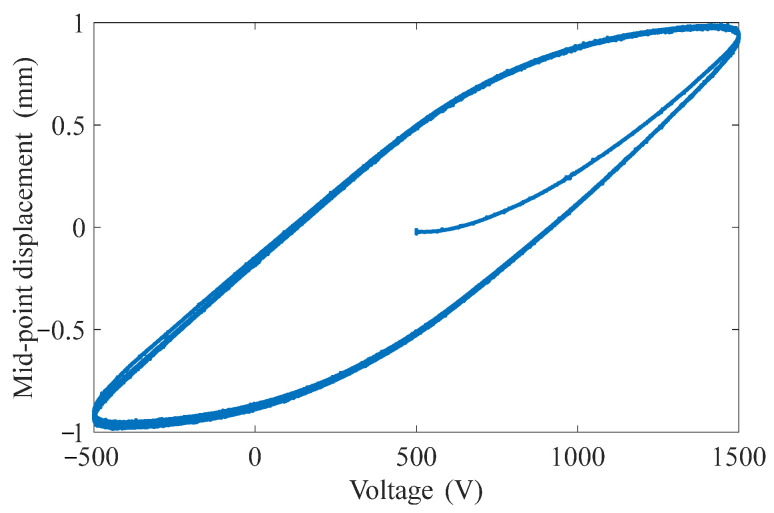
Hysteresis loop of mid-point displacement of MFC-PBP versus quasi-static excitation voltage.

It is noted that (13) is the voltage applied to the one side MFC of the bimorph, and the voltage phase on the other side MFC is 180° different from this voltage phase, so that the maximum deformation of the MFC-PBP can be reached. The hysteresis loops of the mid-point displacement of MFC-PBP versus the quasi-static excitation voltage are shown in Figure 5.

Then, the relative error (RE) is used as the measurement of the prediction accuracy, and its expression is

(14) $$RE=\sqrt{\frac{\sum_{i=1}^{N}{\left(y_{model}^{i}-y_{exp}^{i}\right)}^{2}}{\sum_{i=1}^{N}{\left(y_{exp}^{i}\right)}^{2}}}$$

where the $y_{\mathrm{model}}^{i}$ is the simulation results obtained by the Bouc–Wen model, $y_{\exp}^{i}$ is the test results, and *N* is the sample number.

**Figure 6:**
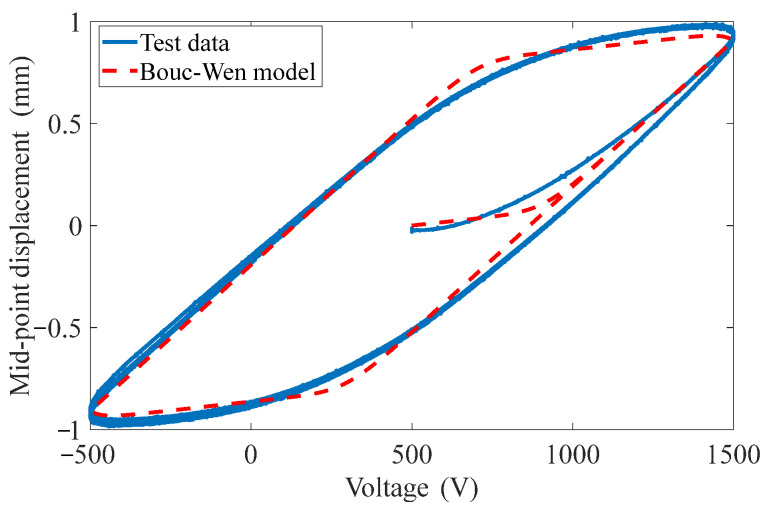
Identified quasi-static hysteresis loop based on Bouc–Wen model.

Taking the RE minimum as the optimization goal, GA is used to identify the model parameters of *d*, *α*, *β*, *γ*, and *n*, and the optimized parameters as listed in Table 1. The identified simulation hysteresis loop is based on the Bouc–Wen model as shown in Figure 6, and the RE of the identified model is 5.99%. It is noted that, since the Bouc–Wen model can only predict the output displacement regarding the symmetric voltage, MFC is driven by an asymmetric voltage of −500 V to +1500 V. Therefore, we first assume an input voltage of −1000 V to +1000 V, and after obtaining the displacement, we add a bias voltage of +500 V to the input voltage, resulting in the hysteresis loop of MFC-PBP, as shown in Figure 6.

Then, the following chirp signal is applied to the obtained Bouc–Wen model, which in the frequency range of 0.25 Hz~1 Hz.

(15) $$\begin{array}{cc} u=1000\sin\left[\pi \left(0.03t+0.5\right)t\right]+500 & 0\leq t<25s \end{array}$$

The output displacement of the Bouc–Wen model is taken as the input of the discrete transfer function, the measured chirp displacement response is considered as the output, the delay orders in the discrete transfer function (3) are set as *n* = 3 and *m* = 2, and then the discrete transfer function can be obtained by least-squares estimation as

(16) $$G\left(z\right)=\frac{2.9870z^{2}-4.9623z+1.9846}{z^{3}-0.6371z^{2}-0.3380z-0.0157}$$

**Figure 7:**
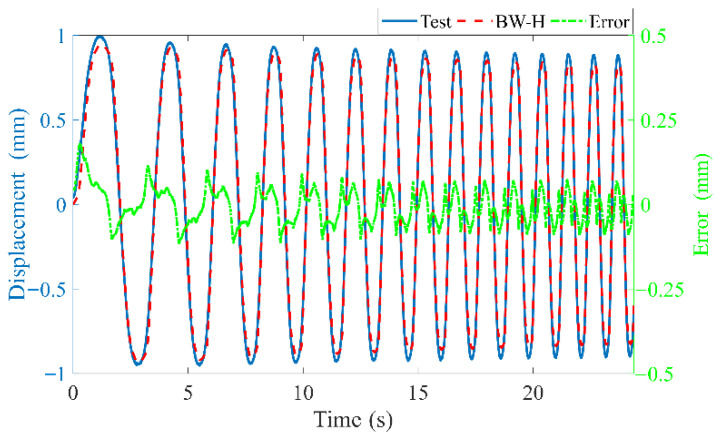
Predicated displacement of the BW-H model under chirp signal.

Thus, the BW-H model is established. Then, the voltage excitation (15) is applied to the BW-H model, and the following results are obtained, as shown in Figure 7. The RE is 7.53% compared with the experimental results. This illustrates that the BW-H model has some prediction ability of rate-dependent hysteresis.

### 3.2. Parameters Identification of LS-SVM Model

**Figure 8:**
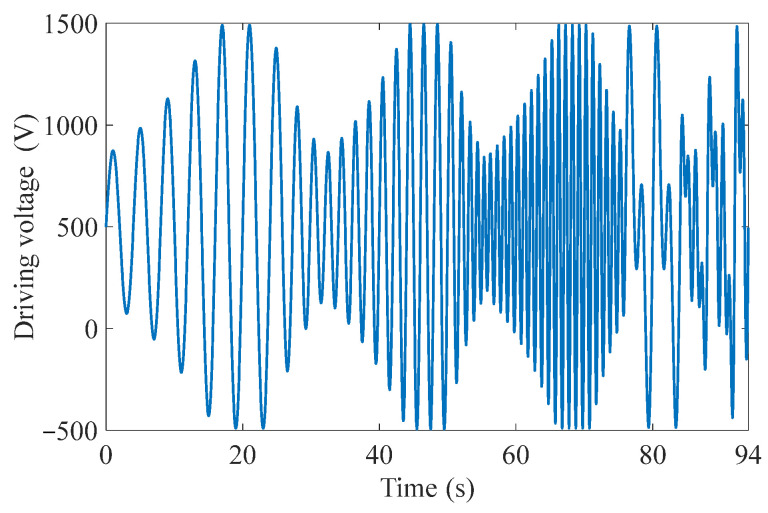
Excitation voltage with variable amplitudes and variable frequencies.

In order to achieve higher driving frequencies away from the resonance frequency of the MFC-PBP, and to improve the generalization capability of the model in various exciting voltages, the LS-SVM model is trained by the following variable voltage. The training voltage includes three frequencies of 0.25 Hz, 0.5 Hz, and 1 Hz, and the amplitude variation range from 350 V to 1000 V, as shown in Figure 8.

**Figure 9:**
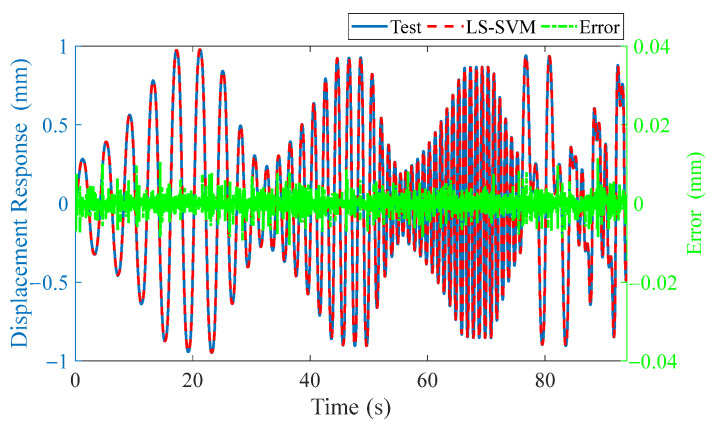
Results comparison of trained LS-SVM and experiment.

Building the input signals as the format in (5), *p* = 3 and *q* = 3 are set. To avoid overfitting, based on 5-fold cross-validation, two hyper-parameters *C* = 8.596 × 10^4^ and *σ* = 0.6019 are obtained, and then the following fitting is realized. The experimental results, training simulation displacement results, and their errors are shown in Figure 9, and the RE is 0.3%.

### 3.3. Generalization Ability Comparison of Two Models

In order to compare the generalization ability of two models, the excitation voltage (17) is used as the generalization ability test signal.

(17) $$\begin{array}{cc} u=440\left[\sin\left(0.5\pi t\right)+\sin\left(\pi t\right)+\sin\left(2\pi t\right)\right]+500 & 0\leq t<30 \end{array}$$

**Figure 10:**
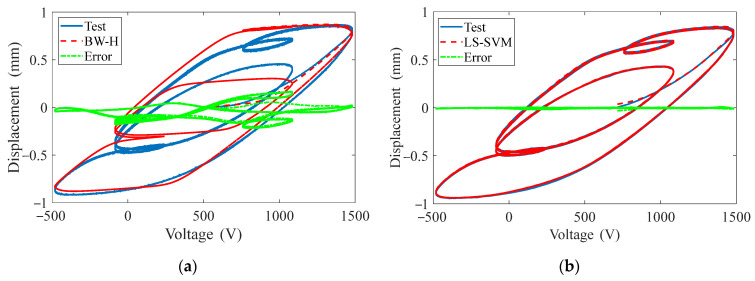
Predicated displacements comparison of two models under the excitation voltage of multiple frequencies superposition: (**a**) BW-H model, (**b**) LS-SVM model.

This signal is applied to the BW-H model and the LS-SVM model to obtain the following results, as shown in Figure 10.

The REs of the BW-H model and the LS-SVM model are 20.9% and 0.76%, respectively. This indicates that the LS-SVM model has much higher prediction accuracy and generalization ability.

## 4. Displacement Tracking Control

The one purpose of the above hysteresis modeling is to obtain the hysteresis characteristic, and the other target is to achieve hysteresis compensation for arbitrary displacement commands. In this section, the hysteresis compensator based on LS-SVM is established, their predicated voltages are compared with those of the inverse model of BW-H, and the inverse model of BW-H refers to Ref. [29]. After that, these two feedforward compensators combine with the PI feedback controller to achieve the displacement tracking for the MFC-PBP, and then the experimental results of these two models are compared.

### 4.1. Driving Voltage Predication

In the hysteresis compensation LS-SVM model, the measured displacement response (Figure 9) and the training voltage signal (Figure 8) are taken as the input and output, respectively. Through many tests, it was found that the input signal consists of the displacement signal, and its differences for the time can obtain better voltage prediction accuracy and generalized ability. The format of input signal is as follows:

(18) $${\tilde{x}}_{k}=\left[y_{k}\;y_{k-1}\ldots y_{k-p}\;{\dot{y}}_{k-1}\ldots {\dot{y}}_{k-q}\right]$$

where ${\dot{y}}_{k-i}=\frac{y_{k-i}-y_{k-i-1}}{\Delta t}$, Δt=0.01 s.

Through 10-fold cross-validation, two hyper-parameters, *C* = 450.5 and *σ* = 0.074, and the predictions of the input voltage are obtained, and compared with the actual training voltage, the RE is 3.04%.

**Figure 11:**
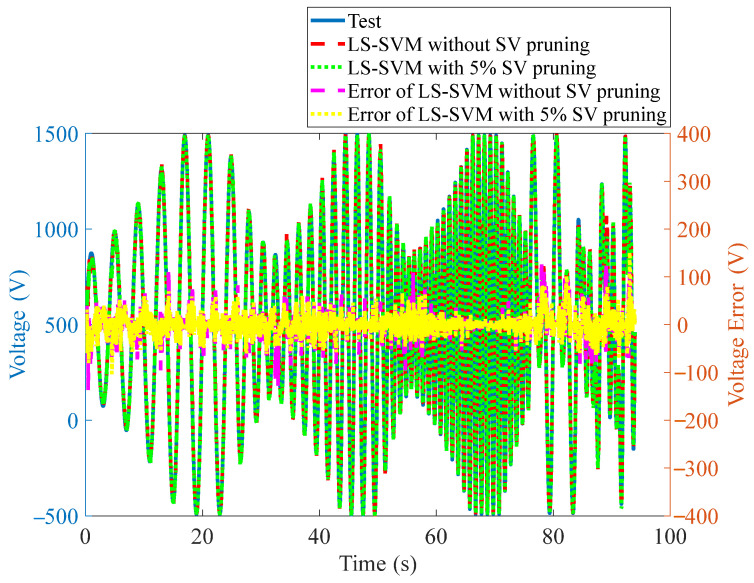
Predicated driving voltages of LS-SVM with and without SV pruning.

The external disturbance and measurement error lead to some outliers in the test results, and the support vectors corresponding to large outliers introduce a greater deviation into the final prediction results; thus, these support vectors need to be pruned. However, pruning too many support vectors will decrease the prediction accuracy, thus the pruning ratio needs to be adjusted. Through many tests, it was found that pruning the support vectors with the largest 5% deviation can predict the driving voltage most accurately. In this case, the two hyper-parameters *C* = 1631.3 and *σ* = 0.1273, the number of support vectors is 8927, and the RE is 2.41%. The voltage prediction results in both cases with and without support vector pruning are shown in Figure 11.

**Figure 12:**
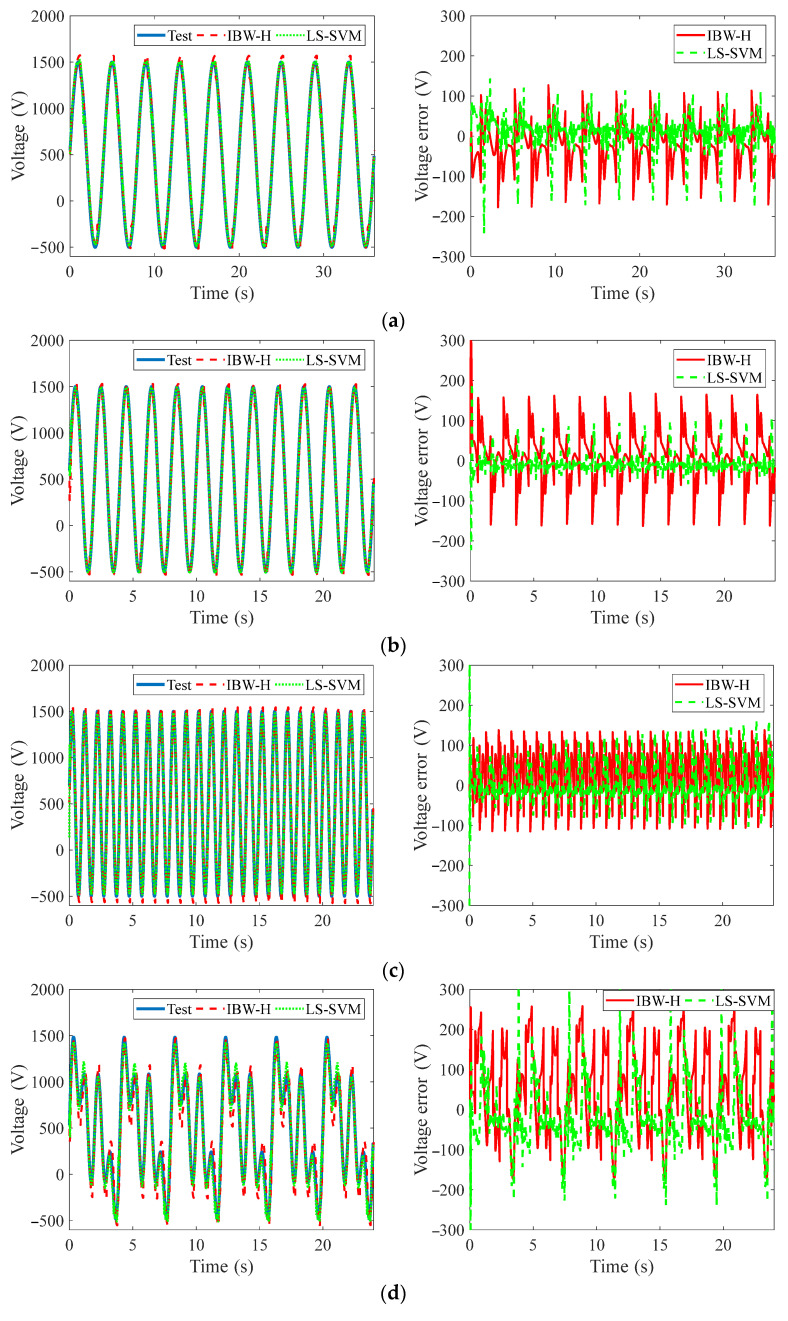

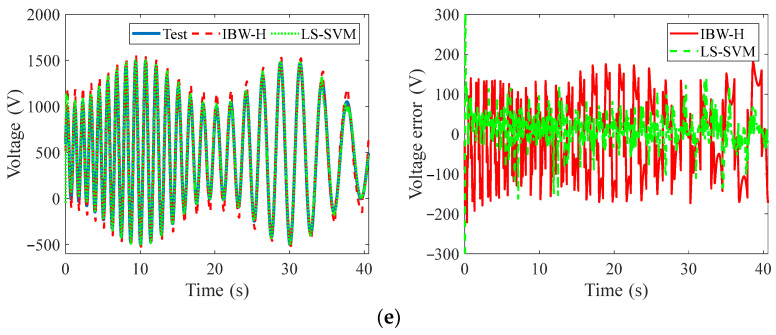
Feedforward voltage predictions of various signals based on hysteresis compensation models of LS-SVM and inverse model of BW-H: (**a**) 0.25 Hz, (**b**) 0.5 Hz, (**c**) 1 Hz, (**d**) 0.25 Hz/0.5 Hz/1 Hz, and (**e**) variable frequency and amplitude.

Based on the hysteresis compensation LS-SVM model with SV pruning and the inverse model of BW-H, the constant frequency, multi-frequency superposition, variable frequency, and amplitude excitation voltages are predicted, and the prediction voltages and their errors are shown in Figure 12a–e, respectively. The prediction REs are listed in Table 2.

By comparing the two model REs, it can be seen that the hysteresis compensator based on the LS-SVM has higher prediction accuracy. Except for the multi-frequency superposition signals, the predicted REs of the LS-SVM model for all signals is less than 6.0%. It is estimated that the large prediction error is mainly caused by the limited size of the training model, which is mainly due to the limited number of support vectors applied to the later online feedforward control. This limitation is determined by the processor and memory sizes of the data acquisition and processing equipment.

### 4.2. Tracking Displacement Control

**Figure 13:**
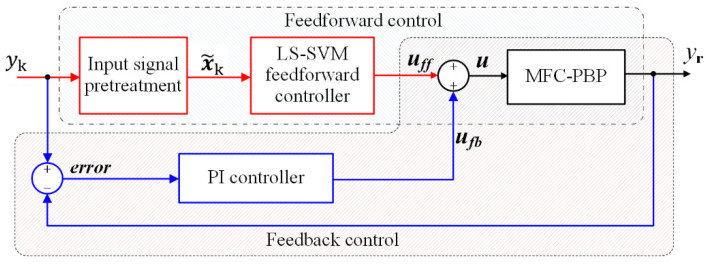
Displacement tracking control diagram of LS-SVM feedforward compensator plus PI feedback control.

Due to the poor anti-disturbance ability of feedforward control, for improving the tracking displacement performance of MFC-PBP, this section conducts the feedforward plus feedback displacement tracking tests for the MFC-PBP under various displacement commands. The hysteresis compensation model of LS-SVM and the inverse model of BW-H model are, respectively, taken as the feedforward compensator, and the PI controller is the feedback controller. The LS-SVM feedforward plus PI feedback displacement tracking control diagram as shown in Figure 13. Through the proportional and integral gains adjusting, we set *K*_p_ = 1, *K*_i_ = 0.32.

**Figure 14:**
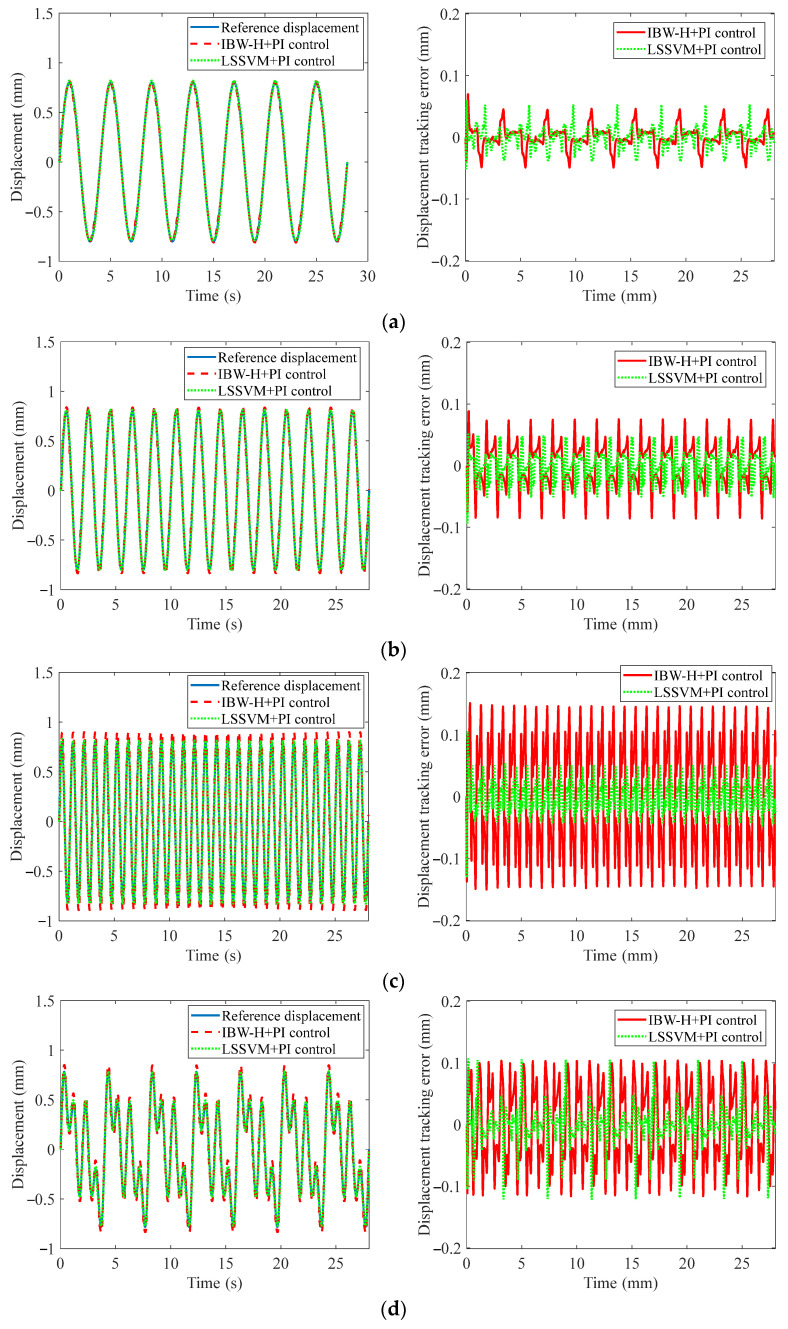

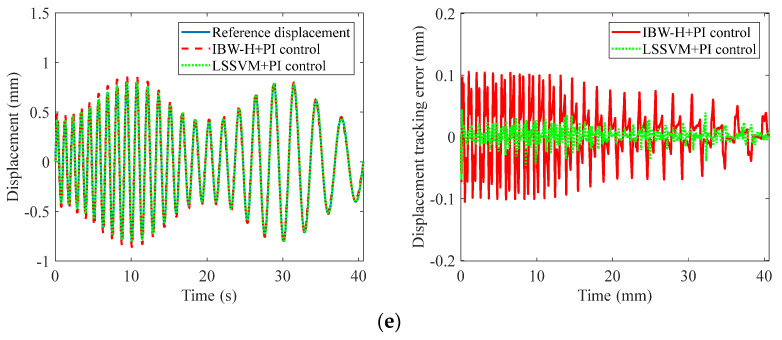
Tracking displacement results of various displacement signals: (**a**) 0.25 Hz, (**b**) 0.5 Hz, (**c**) 1 Hz, (**d**) 0.25 Hz/0.5 Hz/1 Hz, and (**e**) variable frequency and amplitude.

The results of the feedforward plus feedback control abased on two compensators are shown in Figure 14, and the REs of displacements tracking are listed in Table 3.

Figure 14 and Table 3 show that the LS-SVM feedforward compensator combined with PI feedback controller has a higher accuracy than the BW-H compensator with the PI controller. In addition, the REs of the LS-SVM feedforward compensator with PI controller are relatively uniform in various displacement signals, and all REs can be controlled within 8%. However, in the high-frequency case, the REs of the BW-H feedforward compensator combined with the PI controller increase rapidly. Thus, we can draw the conclusion that the feedforward compensator based on the LS-SVM model is more suitable for the displacement tracking control than the BW-H model.

## 5. Conclusions

This paper studies the hysteresis modeling and displacement tracking control for the MFC-PBP by the BW-H model and the LS-SVM model. The simulation and test results show that, compared with the BH-H model, the LS-SVM model has higher precision and the stronger generalization ability in the rate-dependent hysteresis modeling and voltage prediction. In addition, the feedforward plus feedback displacement tracking loop composed by the LS-SVM model and the PI controller also has a higher displacement tracking accuracy than those of the inverse model of BW-H with the PI controller. In the future, a more accurate displacement tracking control strategy for more complex deformation signals needs to be further studied.

## Figures and Tables

**Table 1:** Optimized parameters of Bouc–Wen model.

Parameters	*d*	*α*	*β*	*γ*	*n*
values	1.430 × 10^−^³	1.259 × 10^−^³	0.1631	2.670 × 10^−4^	7.501

**Table 2:** Voltage prediction REs of various signals based on hysteresis compensation models of LS-SVM and inverse model of BW-H.

Cases	0.25 Hz	0.5 Hz	1 Hz	0.25/0.5/1 Hz	Variable Frequency and Amplitude
BW-H	6.28%	7.60%	7.33%	15.85%	12.18%
LS-SVM	4.88%	2.83%	5.63%	11.00%	5.89%

**Table 3:** Tracking displacement REs of various signals.

Cases	0.25 Hz	0.5 Hz	1 Hz	0.25/0.5/1 Hz	Variable Frequency and Amplitude
BW-H+PI	3.07%	6.07%	15.22%	14.75%	8.05%
LS-SVM+PI	2.82%	3.77%	3.81%	7.24%	2.26%

## Data Availability

The data used in this research are properly cited and reported in the main text.
